# Exploring medication adherence in Behçet’s disease following COVID-19: a mixed-methods study

**DOI:** 10.1186/s13023-025-04090-8

**Published:** 2025-11-12

**Authors:** Emily Arden-Close, Fay Sweeting, Danielle Guy, Claudia Mallia, Ala Yankouskaya

**Affiliations:** Bournemouth University, School of Psychology, Poole, BH12 5BB UK

**Keywords:** Behçet's disease, Medication adherence, COVID-19, Mixed methods, Necessity of medicines, Concerns regarding medicines

## Abstract

**Objectives:**

Identify predictors of and explore the decision-making process regarding medication adherence in Behçet’s Disease, a rare autoimmune disease.

**Methods:**

A mixed methods study (cross-sectional plus qualitative) conducted between January and March 2021. In the quantitative study, participants (*n* = 89, age 25–69) completed online questionnaires about demographics, medication adherence (implementation; taking medicine as required), beliefs about medicines, illness perceptions, trust in physicians and fear of COVID-19. Data were analysed using correlations and multiple regressions. In the qualitative study, semi-structured audio interviews were conducted with participants (*n* = 16; age 28–66) via Zoom. Data were analysed using inductive thematic analysis.

**Results:**

Belief in necessity of medicines and concerns about medicines explained 16% of the variance in self-reported adherence to medication, and belief in necessity of medicines explained 24% of the variance in attitude to adherence. Qualitative analysis identified five main themes: experience of illness (excellent understanding of illness, advocating for own care), experience of taking medication (balancing act, part of routine), facilitators to taking medication (doctor recommended, helped symptoms, maintained quality of life), barriers to taking medication (not helping with symptoms, fearing certain medicines, concerns about side effects, difficult to take as prescribed) and additional barriers caused by COVID-19 (fear of COVID-19, concerns medications would increase risk of COVID-19 and decrease response to vaccines, difficulties obtaining medicines).

**Conclusions:**

Adherence to Behçet’s Disease medication was generally good. Concerns participants had about medicines included sometimes not noticing the difference when they stopped taking medicines, fears about some medicines and concerns about side effects. However, based on their excellent understanding of the disease and trust in doctors, these concerns were balanced against the belief that medication was necessary to help their symptoms and maintain quality of life. To address concerns and ensure patients take medicines as prescribed, they need to be provided with up-to-date condition-specific advice.

**Supplementary Information:**

The online version contains supplementary material available at 10.1186/s13023-025-04090-8.

Behçet’s disease is a rare, complex, incurable autoimmune rheumatic condition affecting approximately 1000 people in the United Kingdom and 200,000 in the United States. It causes inflammation of the immune system, manifested in mouth ulcers, joint pain, headaches, gastro-intestinal ulceration, vision-threatening eye inflammation, cognitive impairment, and painful genital ulcers which heal with scarring [1]. Primary treatment consists of oral immunosuppressants, which are often administered alongside the steroid prednisolone. If this fails or the disease is aggressive, biologics such as infliximab or Humira are recommended [2]. Lack of treatment risks flares of the disease, affecting both the patient’s quality of life (QoL) and requiring greater use of healthcare resources [3]. For example, low medication adherence (implementation – the extent to which the patient takes their medication as required) [4] is associated with more oral ulcers in female patients [5]. However, treatment can cause negative side effects such as nausea, weight gain and risk of serious infections [6], and also increase risks of serious complications should the patient develop COVID-19 [7].

Low rates of medication adherence, ranging from 20 to 90%, have been reported in rheumatic diseases, with issues with implementation being prevalent [8, 9]. However, limited research has been conducted on medication adherence in Behçet’s Disease [3, 5, 10, 11]. Prior to COVID-19, greater self-rated adherence was associated with greater belief in the necessity of medicines, and lower self-rated adherence was associated with greater (a) concerns about medicines and (b) beliefs that medicines caused harm, as measured by the Beliefs About Medicines Questionnaire (BMQ) [3]. Since the COVID-19 pandemic started, patients taking immune suppressant medication were recommended to continue with their medication to maintain remission or reduced disease activity [12]. In a cross-sectional study of over 4000 patients with rheumatic disease in Germany, over 90% reported following their rheumatologist’s recommendations to continue immunosuppressant medication [13]. These numbers had scarcely changed relative to pre-pandemic. However, this research asked only one question regarding continued medication adherence. Further exploration of factors influencing adherence is therefore needed.

There is also evidence that trust in healthcare professionals predicts future self-reported medication adherence [14]. Understanding how patients negotiate decision-making regarding their medication could potentially lead to the development of medication-related guidance. Such guidance would be of relevance not only to COVID-19 but could also act as guidance for potential future pandemics. Findings regarding decision-making around medication adherence could also have implications for patients with other similar conditions managed by immunosuppressant medication, such as rheumatoid arthritis [15].

We therefore aimed to conduct a mixed methods study to enhance understanding of the factors affecting medication adherence, specifically implementation, defined as the extent to which the patient takes their medication as required [4], in individuals with Behçet’s Disease living in the UK. In line with Horne’s framework [16], we conceptualised adherence as being influenced by medication-related factors (beliefs in the necessity of and concerns about medicines. Additionally, based on research evidence, we expected that medication adherence would be influenced by the healthcare system (trust in doctors), condition-related factors (disease severity), and patient-related factors (attitude to adherence; fear of COVID-19; emotional representations of the illness). The quantitative data aimed to identify predictors of medication adherence. We hypothesised that medication adherence would be predicted by: beliefs about the necessity of prescribed medicines; concerns about prescribed medicines; belief that medicines are harmful; belief that doctors overuse medicines; trust in healthcare professionals; emotional representations of the illness; fear of COVID-19 and educational level. We also had two mediation hypotheses: (a) that emotional responses to the illness would mediate the relation between symptom severity and adherence to medication and (b) that the relation between fear of COVID-19 and adherence to medication would be moderated by trust in healthcare professionals, such that adherence would be high in individuals with greater trust in their healthcare professional independent of fear of COVID-19, but among individuals with low levels of trust in their healthcare professional, adherence would decrease as fear of COVID-19 increased. The qualitative interviews aimed to explore the experiences of individuals with Behçet’s Disease in more depth, including the decision-making process regarding medication and whether it had changed following COVID-19. Combining the qualitative and quantitative datasets enabled us to examine convergence of the databases, to obtain a more complete understanding of the problem.

## Methods

A convergent mixed methods design (where qualitative and quantitative data provide multiple perspectives on a problem) was used [17, 18]. The quantitative data collection and analysis is presented first, followed by the qualitative data collection and analysis.

### Quantitative data collection

#### Design

The study was a cross-sectional survey administered on Qualtrics™.

#### Participants

Participants were recruited via an advert on a Facebook page for confirmed UK-based Behçet’s Disease patients. FS is a member of the page and had permission to post there for research purposes. Participants were additionally recruited via advertising on social media (Twitter and Facebook). Eligible participants were aged 18 years or over and had a diagnosis of Behçet’s Disease. This meant that they were treated at one of three Behçet’s Disease Centres of Excellence (London, Birmingham, Liverpool), which they visited every 6–12 months [19]. Medication adherence was not an eligibility criterion. Eighty-nine participants were recruited. It was not possible to obtain information about non-responders. The study was conducted from 4th January to 29th March 2021, when the UK was in national lockdown. Approval was received from Bournemouth University Faculty of Science and Technology Research Ethics Committee, ref 34723 on 16th December 2020.

#### Materials

##### Brief illness perception questionnaire (IPQ)

The Brief Illness Perception Questionnaire [20] is a nine-item scale designed to assess cognitive and emotional representations of illness perceptions. The first eight items are scored on a Likert-type scale from 0 to 10, and item 9 involves listing in rank order what participants believe to be the three most important causes of their illness. A higher score indicates a view of the illness as more threatening. It has been used in a variety of illnesses and has high reliability and validity. Cronbach’s alpha in this study was 0.68, indicating satisfactory reliability.

##### Beliefs about medicines questionnaire (BMQ)

The Beliefs about Medicines Questionnaire (BMQ) [21] is an 18-item scale assessed on a 5-point Likert scale from strongly agree to strongly disagree. It consists of two five-item factors assessing beliefs about the necessity of prescribed medication (Specific-Necessity) and concerns about prescribed medication relating to beliefs around long-term toxicity, dangers of dependence and side effects of medicines (Specific-Concerns) and two four-item factors assessing beliefs that medicines are harmful (General-Harm) and beliefs that doctors overuse medicines (General-Overuse). The BMQ was validated on individuals with long-term conditions, including diabetes and kidney disease. It has good psychometric properties, including very good reliability and validity and acceptable test-retest reliability. In this study, Cronbach’s alpha ranged from 0.73 (general overuse subscale) to 0.89 (specific necessity subscale) indicating good reliability.

##### Medication adherence rating scale (MARS)

The Medication Adherence Rating Scale (MARS) [22] is a 10-item scale developed to assess medication adherence in individuals with psychosis. In this study, we utilised the four items related to medication adherence behaviour and four items related to attitude to medication adherence. Previous studies in a variety of chronic conditions, such as rheumatoid arthritis, reported the ability of the MARS questionnaire to measure intentional non-adherence (preference for avoiding, forgetting, adjusting and stopping taking medicines) [23]. In this study, Cronbach’s alpha was 0.61 for adherence behaviour and 0.55 for attitude to adherence, indicating low to acceptable reliability.

##### Trust in physician scale

The Trust in Physician Scale [14] is an 11-item scale assessed on a 5-point Likert scale from 1 (strongly disagree) to 5 (strongly agree). The overall score is obtained by taking the mean of the responses and transforming that into a 0-100 scale. Higher scores indicate higher levels of trust in doctors. Internal consistency in patients with rheumatic disease is high (0.87) [24]. In this study, Cronbach’s alpha was 0.91, indicating excellent reliability.

##### Fear of COVID-19 scale

The Fear of COVID-19 scale [25] was developed in 2020 in Iran in response to the developing COVID-19 pandemic to assess fear of COVID-19. It consists of seven items assessed using a 5-item Likert-type scale from 1 (strongly disagree) to 5 (strongly agree). The validation study demonstrated high internal consistency (0.72) and test-retest reliability (0.82) and good psychometric properties. Cronbach’s alpha in this study was 0.91, indicating excellent reliability.

#### Procedure

After giving consent to participate, participants completed a cross-sectional survey via Qualtrics™ and were debriefed. We collected demographic and medical information about age, gender, length of time living with Behçet’s Disease, time since diagnosis, highest level of education completed and current medications. Questionnaires administered included the Brief Illness Perception Questionnaire (IPQ), Beliefs about Medicines Questionnaire (BMQ), Medication Adherence Rating Scale (MARS), Trust in Physician Scale, and the Fear of COVID-19 scale.

#### Data analysis

Pearson correlations were conducted to explore the relations between the variables. Multiple regression was used to examine the unique contributions of fear of COVID-19, belief in the necessity of medicines for Behçet’s Disease, and concerns about medicines in predicting medication adherence. While Pearson correlations provided an initial overview of bivariate associations, multiple regression was necessary to account for the interrelationships among predictors and to isolate the independent effect of each variable on adherence. This approach allows for a more accurate understanding of how each factor relates to adherence when controlling for the influence of the others. The analysis was further supported by a sensitivity power analysis (G*Power) [26], which confirmed that the sample size (*N* = 89) was sufficient to detect a meaningful change in explained variance (ΔR² = 0.1) with seven predictors (α = 0.05, power = 0.80).

## Results

### Quantitative Results

#### Descriptives

Participants (*n* = 89) were mainly female (87.6%), employed or studying (63.8%), educated to degree level (65.2%), and classified as having moderate severity disease (59.6%). Demographic information is presented in Table 1, mean questionnaire scores in Table 2, and details of medication taken in Supplementary material 1.

**Table 1:** Participant demographic and medical information

Participant characteristics	(*N* = 89)
Age	43.83 (SD = 11.32; range 25–69)
Gender	Female 78 (87.6%)
	Male 11(12.4%)
Employment Status	Employed 53 (59.3%)
	Student 4 (4.5%)
	Retired 5 (5.6%)
	Homemaker 6 (6.7%)
	Unemployed due to ill-health 20 (22.5%)
	Furloughed 1 (1.1%)
Educational level	Postgraduate degree 25 (28.1%)
	Degree 33 (37.1%)
	A-levels or equivalent 20 (22.5%)
	GCSE/vocational qualification 11 (12.4%)
Time since diagnosis	10.89 years (SD = 9.59, range 0–45 years)
Time living with BD before diagnosis	13.2 years (SD = 10.99, range 0–41 years)
Disease classification*	Mild 11 (12.4%)
	Moderate 53 (59.6%)
	Severe 25 (28.1%)

Note. As objective clinical measures of symptom severity were not available due to the UK national lockdown, we provide disease classification based on medications [27]

**Table 2:** Mean scores for the questionnaires

Variable	Score
Trust in doctor	67.29 (SD = 14.36, range 20–98)
Specific necessity	20.09 (SD = 4.11, range 6–25)
Specific concerns	15.23 (SD = 4.17, range 5–25)
Harm	8.93 (SD = 2.69, range = 5–18)
Overuse	10.26 (3.40, range 4–20)
Emotional representations of illness	4.67 (SD = 8.88, range 23–69)
Fear of COVID-19	20.65 (SD = 6.43, range 9–35)
Adherence behaviour	6.90 (SD = 1.19, range 4–8)
Attitude to adherence	6.69 (SD = 1.18, range 4–8)

**Fig. 1:**
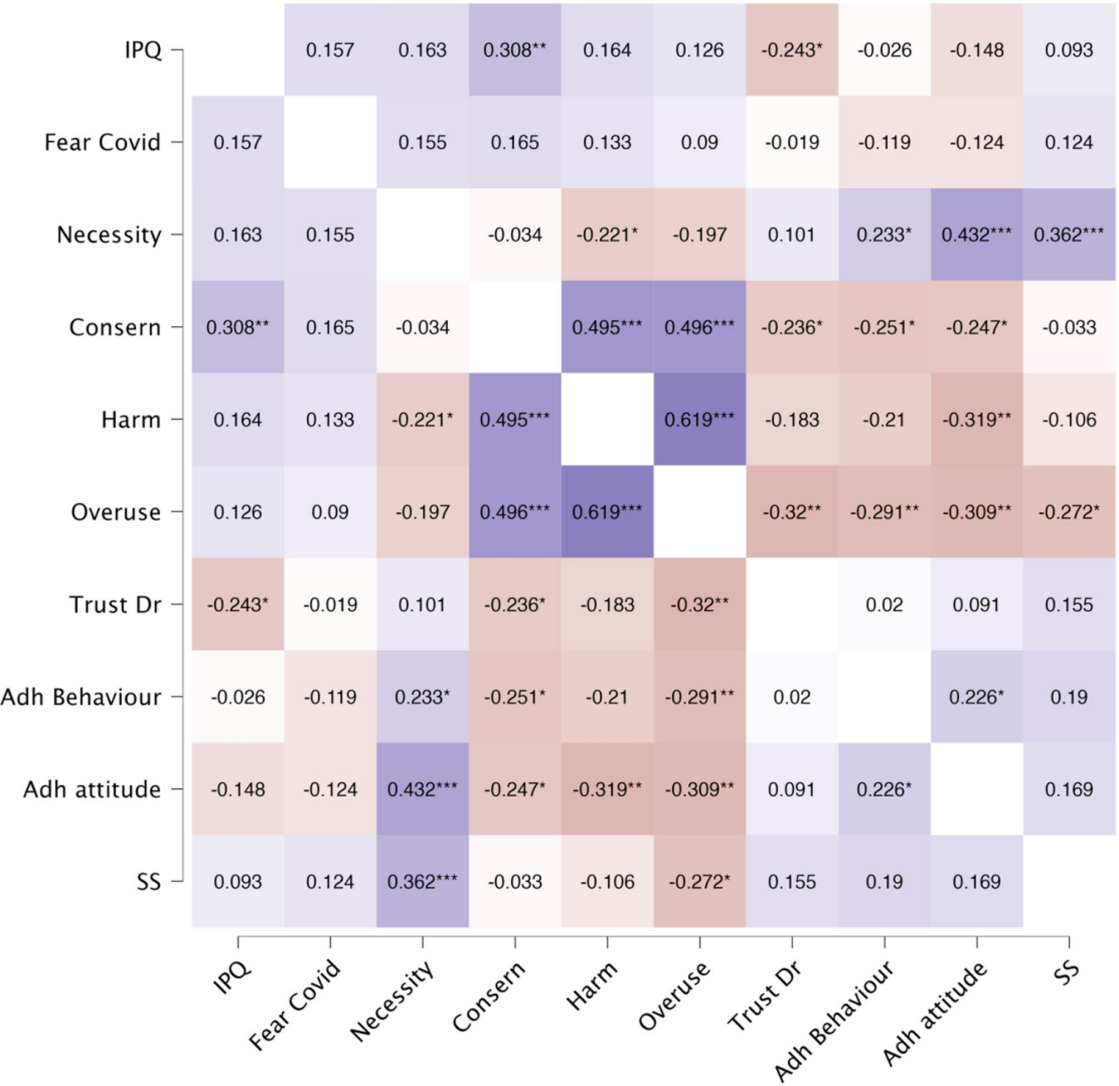
Spearman’s Rho heatmap. * *p* < .05, ** *p* < .01, *** *p* < .001

To gain an initial understanding of the relationship between the variables, we conducted a correlational analysis. Because our variables were measured on ordinal and continuous scales, we used Spearman’s rank-order correlations. The continuous variables were re-expressed as ranked variables (i.e., for each observation, its ordinal rank was compared to the rest of the observations in the sample) making them comparable to the rank of ordinal variables. Results are displayed in Fig. 1 (see details in Supplementary Materials, Table S2).

All subscales of the BMQ (necessity, concern, harm and overuse) were associated with attitude to adherence; and necessity, concern and overuse were also associated with adherence behaviour. Adherence was not associated with attitude and illness representations, trust in doctor, fear of COVID-19, disease severity, age or gender.

To investigate which variables predicted medication adherence, two separate multiple regression analyses were performed using the backward method with alpha level < 0.05. Regression 1 included adherence behaviour as a dependent variable and seven predictors (IPQ, fear of COVID-19, necessity, concern, harm, overuse and trust in doctor). In regression 2, we assessed whether the seven predictors could predict attitude to medication adherence. Prior to entering data into regressions, all variables were scaled. Six potential models were calculated for each regression.

**Fig. 2:**
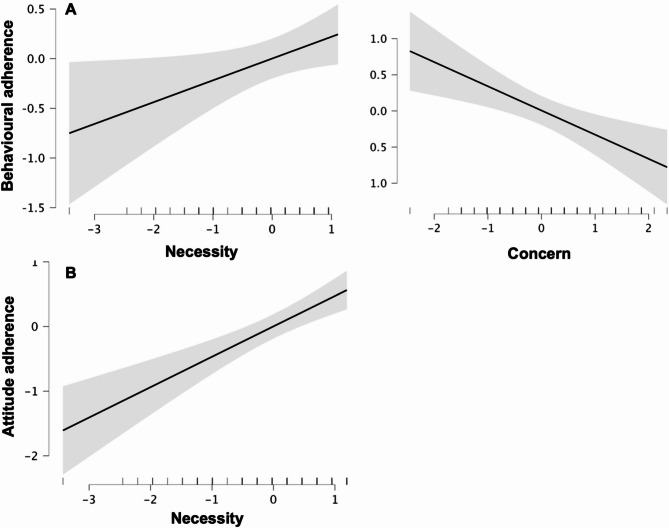
Predictors of adherence behaviour (**A**) and attitude (**B**) to medication. The grey areas represent 95% CIs

The final regression model 1 identified two significant predictors of behavioural adherence to medication: belief in the necessity of medicines and concerns about medicines. Specifically, greater belief in the necessity of medication was associated with higher adherence (B = 0.12, *p* = .03), while greater concerns about medicines were associated with lower adherence (B = -0.33, *p* = .002). Together, these variables explained 16% of the variance in adherence (R² = 0.16), and the model was statistically significant overall (F(2,83) = 8.01, *p* < .001). (Fig. 2A).

In the second regression analysis, belief in the necessity of medicines emerged as the sole significant predictor of attitudes toward adherence to medication. Greater belief is the necessity of medicines was strongly associated with more positive attitudes to adherence (B = 0.47, *p* < .001). This model accounted for 24% of the variance in attitudes (R² = 0.24) and was statistically significant (F(1,84) = 10.26, *p* < .001) (Fig. 2B).

## Methods

### Qualitative Data Collection

#### Procedure

Approval was granted by the Faculty of Science and Technology Ethics committee, ref 34723 on 16th December 2020. Semi-structured audio interviews lasting 19 min to 1 h 17 min (Mean = 50.56 min; SD = 12.84 min) were conducted with 16 individuals with Behçet’s Disease by FS, a researcher with Behçet’s Disease with experience conducting qualitative interviews. Pre-interview, we collected demographic information about age, gender, length of time living with Behçet’s Disease, time since diagnosis, highest level of education completed, and current medications. Interviews covered participants’ experiences of living with Behçet’s Disease, including their experiences and views around taking their medication, and their experiences of managing their condition both prior to and post COVID-19. They were conducted between 5th January and 6th March 2021, when the UK was in national lockdown. Interviews were audio recorded via the videoconferencing platform Zoom and transcribed verbatim.

#### Data analysis

Reflexive thematic analysis (RTA) was conducted, in line with Braun and Clarke’s 2021 guidance [28, 29]. RTA was considered appropriate as it focuses on meaning making, and we aimed to understand how participants made sense of the process of decision-making around medication adherence [28]. Transcriptions were checked for accuracy. Two researchers (EAC and CM) read all transcripts. This led to the development of a coding manual to ensure the data was coded transparently and systematically. All transcripts were coded by EAC (an experienced qualitative researcher), and CM and themes checked with FS. Diagrams were developed to illustrate key themes within the data.

#### Participants

Participants were recruited via a Facebook page for confirmed UK-based Behçet’s Disease patients, on which an advert was placed. FS is a member of the page and had permission to post there for research purposes. Eligible participants were aged 18 years or over and had a diagnosis of Behçet’s Disease. This meant that they were treated at one of three Behçet’s Disease Centres of Excellence (London, Birmingham, Liverpool), which they visited every 6–12 months [19]. Medication adherence was not an eligibility criterion. Participants were purposively sampled to increase gender diversity. There was minimal refusal to participate.

## Results

### Qualitative Results

#### Sample characteristics

Participants ages ranged from 28 to 66 years (Mean = 45.63 years). Participants were mainly female (*n* = 14; 87.5%), white British, employed (*N* = 12, 75%) or studying (*n* = 2, 12.5%). Educational levels ranged from O-levels (*n* = 1; 6.3%) to degree (*N* = 7, 43.8%). Participants had lived with their disease 3–41 years since diagnosis (Mean 13.2) and experienced symptoms 2 months – 33 years (Mean 14.24 years) before diagnosis. Medicines taken included oral immunosuppressants (*n* = 13; 81.3%), steroids (*n* = 8; 50%), biologics (*n* = 8; 50%), pain medication (*n* = 8; 50%), triorasol (*n* = 4; 25%), topical skin creams (*n* = 4, 25%), anticoagulants (*n* = 5; 31.3%); beta blockers (*n* = 3); plasma exchange (*n* = 1; 6.3%) and additional medication such as omeprazole and vitamin D supplements (*n* = 11; 68.8%). For full participant details, see Table S3.

#### Themes identified

**Fig. 3:**
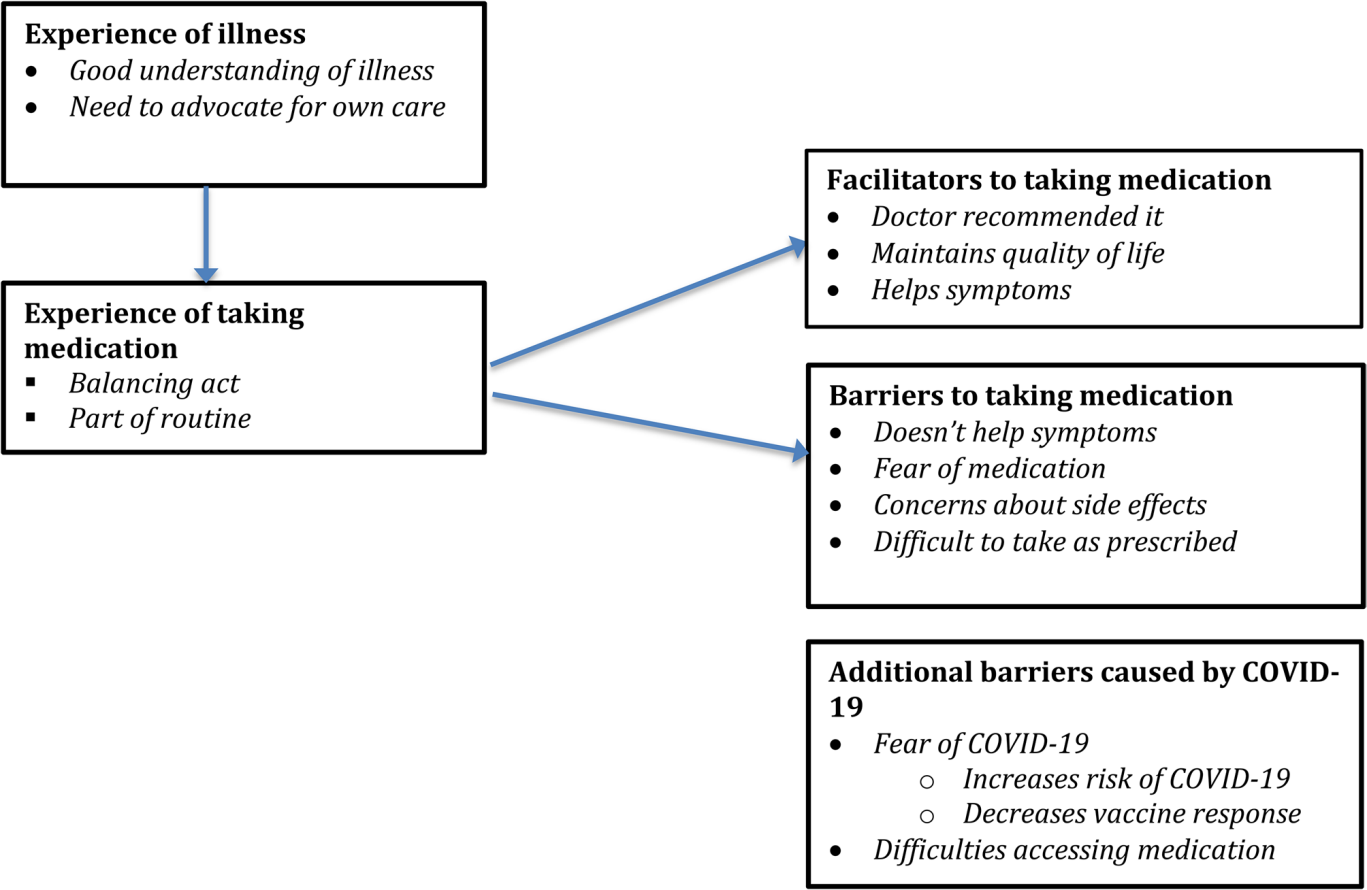
Themes and subthemes identified

Five main themes were identified: Experience of illness, Experience of taking medication, Facilitators to taking medication, Barriers to taking medication and Additional barriers caused by COVID-19. See Fig. 3.

##### Experience of illness

Participants had an **excellent understanding of their illness and medication**. They were extremely well-informed about their condition, being able to describe how their medication worked and why they had been prescribed one medicine rather than another:

So warfarin I was having to go to the doctors every two weeks for an INR test, but with apixaban you don’t need to do that. It works via a different pathway. Whereas Warfarin just thins the blood and hopes for the best, the apixaban actually prevents the clotting process, it disrupts that clotting process. So if a clot starts to form it breaks that down. (P15, male)

This comprehensive knowledge of their illness and medication gave participants the tools and confidence to **advocate for their own care**, which they were often required to do. This involved reminding their doctors to carry out aspects of their care or arguing against coming off a particular medication:

my bloods on azathioprine you’re supposed to have that checked regularly because of liver function issues and my doctors forget it and my consultants get annoyed and then my doctors somehow blame me for that and are like you didn’t get your bloods done. (P8, female)

One participant had even requested to be prescribed medication that was usually available only in the USA, and not prescribed for Behcet’s Disease in the UK:

[medication] That’s the one I take instead of immunosuppressants. It was something that I had done a bit of research about … I spent 18 months harassing my rheumatologist first and eventually she agreed to prescribe it and wrote to my GP … Now at this stage I was on about 17 tablets a day. And I now take the [medication] and an anti-depressant, blood pressure tablets and folic acid and that’s it. (P10, female)

##### Experience of taking medication

Participants saw their experience of taking medication as a **balancing act**. While they mentioned that their medication helped with symptoms and enabled them to maintain quality of life, they also expressed concerns about side effects and longer-term risks: *“I always worried about the long-term risk of taking it [azathioprine] but it was outweighed by the short-term effects of keeping me safe from a flare up.”* (P15, male)

I absolutely hate the thing [Prednisolone] but it just keeps things at bay and obviously with my eyesight. I don’t want to risk any further damage to my eyesight (P16, female)

In general, though, participants saw taking medication as **part of their routine.**

I just got on and took my medication, I didn’t really think about it. My son learnt to count by counting my tablets every morning, lunchtime and evening. You know two yellows, two blues, two white, and how many tablets have we got here. (P13, female)

##### Facilitators to taking medication

Participants reported taking their medication because their **doctor recommended it.** They assumed their doctor knew what was best for them, so did as they were told.

I think for me it was just more … a sort of given that actually if I’m feeling ill and doctors are recommending take something for it. Then I’ll take something for it and sort of assume that they have more knowledge than I do about these things. (P6, female)

Nevertheless, all but one of the participants felt their medication **helped their symptoms**, consistently reporting that symptoms returned quickly if they stopped taking it. Although some participants disliked the side effects of their medication, they continued to take it as it kept their symptoms under control.

I know if I’ve missed them [colchicine] accidentally and always take them in the morning. And if I miss them then I know I can have them [ulcers] by the afternoon. That’s how fast it goes. (P11, female)

Prednisolone, that’s the steroid that helps with inflammation in the body and it's like a wonder drug. It’s also got horrible side effects, which I hate but it is the one thing that when I’m falling apart will stop Behçet’s in its tracks and makes everything so much better. (P5, female)

Some participants reported having to wait fixed intervals between treatments (such as plasma exchange), which sometimes took place in a hospital. During these intervals, they noticed when they felt they needed a new treatment:

Currently, my memory is very up and down anywhere. I am waiting for the next plasma exchange. About a few days after I have had plasma exchange I’m brilliant again up to my level of brilliance for four/five weeks then it tails off and stuff comes back again. (P3, female)

By **helping their symptoms**, participants felt medication enabled them to **maintain quality of life**, so they could carry out everyday tasks such as working, caring for children and family members, exercising, and socialising. Before receiving medication, they reported being in and out of hospital, experiencing almost unbearable pain and constantly being exhausted:

Without the medication I literally wouldn’t be able to function. … I was in and out of hospital all the time just with pain being excruciating, ulceration being unmanageable so to be honest the medication does give me a quality of life. (P1, female)

It is literally a lifeline. Without that medication I am bed bound, house bound, dead bound … without it, I think I probably would commit suicide to be honest. (P12, female)

##### Barriers to taking medication

On the other hand, participants mentioned several barriers to taking their medication. Some felt their medication **did not help with symptoms**:

Apparently it [Humira] was [helping my symptoms] because my level or my blood tests indicated that it was, but I honestly didn’t feel any different (P4, female)

Some participants also reported not noticing any difference when they missed doses or stopped taking their medication. However, the majority reported not missing doses for longer than a week or two at most:

[If I didn’t take] Colchicine um, probably not a huge not massive impact. Because I have had to stop taking them for a couple of weeks before, and I was fine. (P3, female)

Although rare, some participants mentioned that they **feared taking certain medications**, either because of stories they had heard or because they had reacted negatively to previous prescriptions. For our participants, though, these fears were not sufficient to stop them from taking the medicines:

I think the first dose I had of campath I almost said that I wasn’t gonna go through with it because I read so many horror stories with it but I’m glad I did because it wasn’t as bad … I got a bit of a rash and bit of sickness. That was it (P9, female)

At the beginning I was anxious [about taking Imraldi] because I had already had a massive reaction to Humira (P12, female)

Many participants expressed **concerns about side effects** of their medication. Such concerns mainly related to steroid medication and biologics. On balance, participants strongly disliked taking these medicines, but considered them a necessary evil for managing symptoms:

… these drugs all of them, the biologics everything are dangerous … There are three pages of side effects for the drug. I’ve never seen a list of side effects that long. (P7, female)

they [oral steroids] are good but then you find like that the higher the dose the more side effects you get and also with the reflux and finding that it makes it a lot worse. (P9, female)

Some participants mentioned that although they understood the importance of their medication and wished to take it, it was **difficult to take as prescribed**, due to restrictions on eating or drinking around doses:

I have to do it [magic mouthwash] four times a day, which is really annoying… You’ve got to do it after you’ve eaten or before but a long time before because once you’ve taken it you can’t drink or eat for like an hour (P5, female)

##### Additional barriers to taking medication post COVID-19

From the start of the first UK lockdown in March 2020, most individuals with Behçet’s Disease were classified as clinically extremely vulnerable and advised to shield due to the immune suppressant medication they were taking. Unsurprisingly, most participants reported considerable **fear of COVID-19**:

[My risk of COVID is] Extremely high I’m convinced if I get it I will end up in hospital. Well, that’s if there’s any room in the hospital. I’m really frightened. (P4, female)

far as I understand it I’m clinically extremely vulnerable and I am to be shielding at the moment. And if I caught covid I would most likely have complications. (P5, female)

This fear came from two concerns. First, most participants expressed concerns that their medication would **increase their risk of COVID-19.** Participants had been told by doctors that their medication made them at greater risk of complications if they developed COVID-19, and even that there was a real risk of them not surviving if they developed COVID-19:

Having Behçet’s itself doesn’t mean that I’ll catch covid any quicker but taking the medication I do means that I’m much more vulnerable to not being alive if I get Covid which is really frightening when a doctor tells you that down the phone. It’s really frightening to hear them say I can’t guarantee that you will survive this. (P2, female)

Although most participants still took their medication as prescribed, many reported a shift in their attitude to it. Medication that had previously enabled them to live full lives was now restricting their lives, causing them to hesitate more before taking it: *“… how I take my medication hasn’t really changed. It’s just my thought process around it has changed”* (P2, female). In a few cases, this fear even led some participants to stop taking their medication or at least become less adherent to it: *“I do try and skip the odd day. So whereas before covid I was very rigid with my meds*,* during lockdown I haven’t been.”* (P16, female).

Participants also expressed concerns that their medication might **decrease their response to the COVID-19 vaccination.** First, some medicines taken for Behçet’s Disease potentially block production of antibodies in response to vaccination, and second, as the vaccine had not been tested on immunocompromised individuals, there was no way for participants were to determine whether it would be effective for them. This did not discourage participants from taking vaccinations they were offered, but it did mean they were concerned about how much protection they would have against COVID-19 post-vaccination.

What they can’t tell us is if we will produce the antibodies needed to protect us from covid … It’s [vaccine] come with a little bit of hesitancy or a little bit of caution because I know that the medication I’m on blocks that inflammatory response that the vaccine is trying to stimulate and also that the medication I’m on also blocks production of antibodies. (P2, female)

Some participants experienced **difficulties accessing medication**, which were exacerbated by the COVID-19 pandemic. This was partly due to medical appointments being cancelled during lockdowns, which meant patients were unable to obtain the repeat prescriptions they needed. Participants were concerned their medication would run out, which had happened during earlier lockdowns, leading to their condition worsening.

in 2020 in August, I was really poorly and I didn’t actually get seen until October and I didn’t get the medication I needed til November by which point I was in a really bad state. (P5, female)

There was a period of time where I couldn’t get hold of the dexamethasone eye drops because I think they were starting to use them as treatment [for COVID-19]. (P11, female)

## Discussion

This mixed methods study aimed to enhance understanding of the factors influencing medication adherence in Behçet’s Disease, based on insights from both quantitative and qualitative research. Integration of the qualitative and quantitative findings [18] is presented in Table 3, followed by a discussion of the findings in relation to previous research.

**Table 3:** Joint display of qualitative and quantitative findings and mixed methods meta-inferences

Quantitative Findings	Qualitative Findings	Mixed methods meta-inferences
Greater belief in the necessity of medications was associated with a more positive attitude to adherence and better adherence	Participants had an excellent understanding of their condition and ability to advocate for their own care Participants were aware that medicines would alleviate their symptoms and improve QoL Participants saw taking medicines as part of their routine	The qualitative findings demonstrated the complex decision-making process Behçet’s patients experience; they saw taking medicines as a balancing act between maintaining QoL and experiencing side effects
More concerns about medicines were associated with poorer adherence	Some participants reported concerns that medicines did not help with symptoms. Despite reporting concerns about side effects, participants continued to take medicines to maintain QoL	Concerns about medicines are a barrier to adherence. The main concern preventing adherence is that medicines do not help with symptoms
Trust in doctors was not associated with adherence or attitude to adherence	Participants had a high level of trust in their specialist doctors	The quantitative findings were likely due to a ceiling effect
Illness representations were not associated with adherence or attitude to adherence	Although some participants reported fears of some treatments these were not a barrier to adherence	Participants trusted their doctors, so followed their recommendations
Fear of COVID-19 was not associated with adherence or attitude to adherence	Participants reported fears that immunosuppressant medication might (a) increase their risk of complications should they develop COVID-19 and (b) decrease COVID-19 vaccine response, but continued to value their medication for maintaining QoL	Participants were aware of the importance of their medication for maintaining quality of life, despite concerns; following medical advice may be a greater issue in individuals with low educational levels
	Lack of medication supply was a problem for a significant minority following COVID-19	Lack of supply may also be a barrier to medication adherence

Our quantitative findings revealed that stronger belief in the necessity of prescribed medicines and fewer concerns about negative side effects were associated with more positive attitudes to adherence to medication and better adherence, in line with previous Behçet’s Disease research [3]. The qualitative data highlighted the complex decision-making process Behçet’s patients experience to maintain QoL. Adding to the quantitative findings, it revealed participants’ excellent understanding of their condition and ability to advocate for their own care. This was underpinned by knowledge that their medicines would generally alleviate their symptoms and improve their quality of life in line with doctors’ recommendations, but were also likely to cause side effects. Additionally, it revealed that participants saw taking their medicines as part of their routine. These findings reinforce the importance of clear doctor-patient communication around medication adherence [30].

Adherence was not quantitatively associated with trust in doctors. However, the qualitative findings revealed that good medication adherence (implementation) likely related to positive experiences with specialist physicians, leading to high levels of trust with healthcare professionals, plus the development of a regular routine. Even when participants feared taking medicines, they trusted their doctors and were rewarded with improved QoL. Similar levels of patient satisfaction with specialised centres have been reported in research on other rare diseases [31]. The low correlation between trust in doctor and medication adherence may be due to a ceiling effect regarding trust in doctors. Alternatively, participants may have completed the Trust in Physicians scale with trust in general practitioners in mind – the scale does not specify which doctor to consider. Patients with Behçet’s Disease often take many years to receive a diagnosis, which has led to reduced trust in doctors in other rare diseases e.g [32].

The quantitative study found that more concerns about negative side effects were associated with poorer medication adherence. Similarly, in the qualitative study, participants reported barriers to taking medication such as concerns that they did not help with symptoms, and concerns about side effects, although the main reason given for skipping medication doses was concerns that medicines did not help with symptoms. Concerns about side-effects have been associated with poorer adherence in other long-term illnesses e.g [33]. Interestingly, in the quantitative study, emotional representations of the illness were not associated with adherence. The qualitative study revealed that although some participants had initial fears about taking some medicines, these were soon overcome by the impact of the medicines on their QoL.

The quantitative study found that adherence was unrelated to fear of COVID-19. However, the qualitative study revealed additional barriers to adherence following COVID-19. Participants understood that immunosuppressant medication might both increase their risk of complications should they develop COVID-19 and decrease COVID-19 vaccine response, in line with research showing that use of immunosuppressant drugs has been associated with higher perception of risk from COVID-19 [34]. Nevertheless, participants reported that these barriers did not influence their decision to take their prescribed medication, but rather their attitude to it. At the same time, participants continued to value their medication for maintaining quality of life, in line with recent findings that patients with rheumatic disease continued to heed doctors’ recommendations to take their immunosuppressant medication following the COVID-19 pandemic [13]. This finding may explain the lack of correlation between attitude to adherence and fear of COVID-19. However, this issue needs further exploration with individuals of a lower educational level, as less education is associated with poorer understanding of medicines [35].

Regarding vaccination, at the time of the study (early 2021), clear guidance was not available as COVID-19 vaccines had not been tested on immunocompromised individuals. Given evidence that vaccination reduces risk of SARS-Cov-2 infection in patients taking immunosuppressant medication [36], and that severe vaccine-related adverse events are low [37], the overall advice to patients on immunosuppressant medication is that the benefits of the vaccine outweigh risks of side effects [38]. However, given the balancing act in Behçet’s Disease of taking medicines to manage symptoms under the constant shadow of side effects and long-term health risks, it is critical that patients are provided with condition-specific up-to-date information to enable them to continue to both advocate for themselves and seek advice from medical professionals should they have specific concerns regarding their medicines and COVID-19 vaccines. This issue remains important as first, individuals with Behçet’s Disease (and other diseases requiring immune suppressant medication) are likely to be offered booster vaccinations for years to come, and second, future pandemics might necessitate the development of further vaccines.

### Strengths and limitations

This is one of the first studies to explore medication adherence in Behçet’s Disease, and the first to do so in a UK context. It offers a more in-depth analysis of the decision-making process around taking immunosuppressant medication relative to previous studies (e.g [13]). The mixed methods approach enabled us to gain a more complete understanding of medication adherence among this population than would have been obtained from quantitative or qualitative data alone. Interviews were conducted and recruitment facilitated by a researcher with lived experience of Behçet’s Disease (FS), which enabled in-depth insight into participants’ experiences (particularly helpful with a rare disease). FS was able to build a good rapport with participants [39], which facilitated collection of rich data. To maintain objectivity, the analysis was conducted by an experienced qualitative researcher from outside the target population.

The study has several limitations. Despite our efforts to sample for diversity, most participants were female, almost all were white British, and individuals with lower educational levels were under-represented. These points are important as male participants in the qualitative study reported less time from symptoms to diagnosis than female participants, and medication adherence may be lower in ethnic minorities and individuals of low SES [40]. Although the questionnaire used to assess adherence behaviour (implementation) and attitude to adherence assessed both intentional and nonintentional adherence, it had low reliability and was assessed using yes/no questions. A Likert-type scale might have been better placed to explore complexities in medication adherence. We did not have access to medical records, meaning that first, it was not possible to assess disease severity with clinical precision and second, reports of both prescribed medication and adherence were subject to bias. However, participants appeared to have an excellent understanding of their condition and how their medicines worked, which mitigated this risk. Finally, in convergent mixed methods studies, both forms of data should be collected using the same constructs/concepts and using similar sample sizes [18]. While the quantitative survey and the qualitative interviews addressed the same constructs, the sample sizes differed considerably. However, we argue that as the intent of qualitative and quantitative research differs (qualitative research enables an in-depth perspective [28], whereas quantitative research aims to generalise to the wider population), each sample provides a sufficient account of the data in question.

## Conclusions

Adherence to Behçet’s disease medication is generally good and did not change following COVID-19. This high level of adherence is related to trust in specialist doctors and very good knowledge of the impact of both the illness and their medicines on their bodies. Although participants saw implementation of medication adherence as a balancing act due to fears about certain medicines, concerns about side effects (mainly related to steroid medications) and sometimes not noticing the difference if they stopped taking medicines, they continued to take their medicines. This was because they were aware that medicines reduced their symptoms and enabled them to maintain quality of life. To address medication-related concerns and ensure patients continue to take their medicines as prescribed, it is essential they are provided with up-to-date condition-specific advice.

## Supplementary Information

Below is the link to the electronic supplementary material.

Supplementary Material 1

Supplementary Material 2

Supplementary Material 3

## Data Availability

The datasets generated during and/or analysed during the current study are not publicly available to maintain participant privacy but are available from the corresponding author on reasonable request.
