# Supplementary material for: Exploring medication adherence in Behçet’s disease following COVID-19: a mixed-methods study
Source: Orphanet J Rare Dis. 2025 Nov 12;20:577. doi: 10.1186/s13023-025-04090-8 (PMC12613492; doi:10.1186/s13023-025-04090-8)
Supplement: Supplementary file 2 — Supplementary Material 2 [file 13023_2025_4090_MOESM2_ESM.docx]

**Supplementary Materials**

Table S2.

| Spearman’s correlation between variables. Confidence intervals based on 1000 bootstrap replicates. | | | | | | | | | | | | | | | | | | | | | | | |
| --- | --- | --- | --- | --- | --- | --- | --- | --- | --- | --- | --- | --- | --- | --- | --- | --- | --- | --- | --- | --- | --- | --- | --- |
| **Variable** | |  | | **IPQ** | | **Fear Covid** | | **Necessity** | | **Consern** | | **Harm** | | **Overuse** | | **Trust Dr** | | **Adh Behaviour** | | **Adh attitude** | | **SS** | |
| 1. IPQ |  | Spearman's rho |  | — |  |  |  |  |  |  |  |  |  |  |  |  |  |  |  |  |  |  |  |
|  |  | p-value |  | — |  |  |  |  |  |  |  |  |  |  |  |  |  |  |  |  |  |  |  |
|  |  | Upper 95% CI |  | — |  |  |  |  |  |  |  |  |  |  |  |  |  |  |  |  |  |  |  |
|  |  | Lower 95% CI |  | — |  |  |  |  |  |  |  |  |  |  |  |  |  |  |  |  |  |  |  |
| 2. Fear Covid |  | Spearman's rho |  | 0.157 |  | — |  |  |  |  |  |  |  |  |  |  |  |  |  |  |  |  |  |
|  |  | p-value |  | 0.148 |  | — |  |  |  |  |  |  |  |  |  |  |  |  |  |  |  |  |  |
|  |  | Upper 95% CI |  | 0.370 |  | — |  |  |  |  |  |  |  |  |  |  |  |  |  |  |  |  |  |
|  |  | Lower 95% CI |  | -0.078 |  | — |  |  |  |  |  |  |  |  |  |  |  |  |  |  |  |  |  |
| 3. Necessity |  | Spearman's rho |  | 0.163 |  | 0.155 |  | — |  |  |  |  |  |  |  |  |  |  |  |  |  |  |  |
|  |  | p-value |  | 0.132 |  | 0.154 |  | — |  |  |  |  |  |  |  |  |  |  |  |  |  |  |  |
|  |  | Upper 95% CI |  | 0.350 |  | 0.364 |  | — |  |  |  |  |  |  |  |  |  |  |  |  |  |  |  |
|  |  | Lower 95% CI |  | -0.041 |  | -0.047 |  | — |  |  |  |  |  |  |  |  |  |  |  |  |  |  |  |
| 4. Consern |  | Spearman's rho |  | 0.308 | ** | 0.165 |  | -0.034 |  | — |  |  |  |  |  |  |  |  |  |  |  |  |  |
|  |  | p-value |  | 0.004 |  | 0.130 |  | 0.758 |  | — |  |  |  |  |  |  |  |  |  |  |  |  |  |
|  |  | Upper 95% CI |  | 0.500 |  | 0.368 |  | 0.182 |  | — |  |  |  |  |  |  |  |  |  |  |  |  |  |
|  |  | Lower 95% CI |  | 0.101 |  | -0.049 |  | -0.244 |  | — |  |  |  |  |  |  |  |  |  |  |  |  |  |
| 5. Harm |  | Spearman's rho |  | 0.164 |  | 0.133 |  | -0.221 | * | 0.495 | *** | — |  |  |  |  |  |  |  |  |  |  |  |
|  |  | p-value |  | 0.130 |  | 0.222 |  | 0.040 |  | < .001 |  | — |  |  |  |  |  |  |  |  |  |  |  |
|  |  | Upper 95% CI |  | 0.378 |  | 0.351 |  | -0.015 |  | 0.653 |  | — |  |  |  |  |  |  |  |  |  |  |  |
|  |  | Lower 95% CI |  | -0.058 |  | -0.094 |  | -0.414 |  | 0.293 |  | — |  |  |  |  |  |  |  |  |  |  |  |
| 6. Overuse |  | Spearman's rho |  | 0.126 |  | 0.090 |  | -0.197 |  | 0.496 | *** | 0.619 | *** | — |  |  |  |  |  |  |  |  |  |
|  |  | p-value |  | 0.246 |  | 0.408 |  | 0.067 |  | < .001 |  | < .001 |  | — |  |  |  |  |  |  |  |  |  |
|  |  | Upper 95% CI |  | 0.342 |  | 0.317 |  | 0.028 |  | 0.655 |  | 0.749 |  | — |  |  |  |  |  |  |  |  |  |
|  |  | Lower 95% CI |  | -0.093 |  | -0.132 |  | -0.416 |  | 0.298 |  | 0.453 |  | — |  |  |  |  |  |  |  |  |  |
| 7. Trust Dr |  | Spearman's rho |  | -0.243 | * | -0.019 |  | 0.101 |  | -0.236 | * | -0.183 |  | -0.320 | ** | — |  |  |  |  |  |  |  |
|  |  | p-value |  | 0.024 |  | 0.864 |  | 0.355 |  | 0.029 |  | 0.091 |  | 0.003 |  | — |  |  |  |  |  |  |  |
|  |  | Upper 95% CI |  | -0.035 |  | 0.204 |  | 0.306 |  | -0.044 |  | 0.028 |  | -0.110 |  | — |  |  |  |  |  |  |  |
|  |  | Lower 95% CI |  | -0.433 |  | -0.240 |  | -0.121 |  | -0.413 |  | -0.395 |  | -0.521 |  | — |  |  |  |  |  |  |  |
| 8. Adh Behaviour |  | Spearman's rho |  | -0.026 |  | -0.119 |  | 0.233 | * | -0.251 | * | -0.210 |  | -0.291 | ** | 0.020 |  | — |  |  |  |  |  |
|  |  | p-value |  | 0.812 |  | 0.273 |  | 0.030 |  | 0.019 |  | 0.050 |  | 0.006 |  | 0.855 |  | — |  |  |  |  |  |
|  |  | Upper 95% CI |  | 0.181 |  | 0.111 |  | 0.428 |  | -0.045 |  | 0.010 |  | -0.077 |  | 0.223 |  | — |  |  |  |  |  |
|  |  | Lower 95% CI |  | -0.236 |  | -0.339 |  | 0.028 |  | -0.459 |  | -0.426 |  | -0.489 |  | -0.186 |  | — |  |  |  |  |  |
| 9. Adh attitude |  | Spearman's rho |  | -0.148 |  | -0.124 |  | 0.432 | *** | -0.247 | * | -0.319 | ** | -0.309 | ** | 0.091 |  | 0.226 | * | — |  |  |  |
|  |  | p-value |  | 0.170 |  | 0.257 |  | < .001 |  | 0.021 |  | 0.003 |  | 0.004 |  | 0.403 |  | 0.035 |  | — |  |  |  |
|  |  | Upper 95% CI |  | 0.056 |  | 0.092 |  | 0.592 |  | -0.038 |  | -0.121 |  | -0.100 |  | 0.284 |  | 0.424 |  | — |  |  |  |
|  |  | Lower 95% CI |  | -0.359 |  | -0.330 |  | 0.228 |  | -0.422 |  | -0.499 |  | -0.485 |  | -0.107 |  | 0.016 |  | — |  |  |  |
| 10. SS |  | Spearman's rho |  | 0.093 |  | 0.124 |  | 0.362 | *** | -0.033 |  | -0.106 |  | -0.272 | * | 0.155 |  | 0.190 |  | 0.169 |  | — |  |
|  |  | p-value |  | 0.389 |  | 0.257 |  | < .001 |  | 0.761 |  | 0.326 |  | 0.011 |  | 0.154 |  | 0.079 |  | 0.119 |  | — |  |
|  |  | Upper 95% CI |  | 0.304 |  | 0.320 |  | 0.544 |  | 0.194 |  | 0.102 |  | -0.084 |  | 0.343 |  | 0.381 |  | 0.384 |  | — |  |
|  |  | Lower 95% CI |  | -0.130 |  | -0.125 |  | 0.169 |  | -0.231 |  | -0.316 |  | -0.446 |  | -0.052 |  | -0.004 |  | -0.060 |  | — |  |
|  | | | | | | | | | | | | | | | | | | | | | | | |
| * p < .05, ** p < .01, *** p < .001 | | | | | | | | | | | | | | | | | | | | | | | |
|  | | | | | | | | | | | | | | | | | | | | | | | |
