# Supplementary material for: Exploring medication adherence in Behçet’s disease following COVID-19: a mixed-methods study
Source: Orphanet J Rare Dis. 2025 Nov 12;20:577. doi: 10.1186/s13023-025-04090-8 (PMC12613492; doi:10.1186/s13023-025-04090-8)
Supplement: Supplementary file 3 — Supplementary Material 3 [file 13023_2025_4090_MOESM3_ESM.docx]

Table S3: Medication taken by participants in the qualitative study

| **Gender** | **Age** | **Time since diagnosis** | **Time living with BD before diagnosis** | **Medication*** | **Disease Severity**** |
| --- | --- | --- | --- | --- | --- |
| Female | 28 | 8 years | Since age 13 | Oral immunosuppressants; Biologic; Pain; Anticoagulant/blood thinners | Severe |
| Female | 40 | 17 years | 14 months | Oral immunosuppressants; Biologic; Steroid; Additional; Betablockers | Severe |
| Female | 45 | 17 years | 15 years | PLEX; Steroid; Pain; Additional; Anticoagulant/blood thinners; Topical | Severe |
| Female | 51 | 6 years | 25 years | Oral immunosuppressants; Pain; Additional; MM; Biologic | Moderate |
| Female | 37 | 3 years | 19 years | Biologic; Oral immunosuppressants; Anticoagulant/blood thinner; Pain  Additional; MM; Topical | Severe |
| Female | 39 | 9 years | 3-5 years | Biologic; Oral immunosuppressant; Pain; MM; Steroid; Additional | Severe |
| Female | 59 | 18 months | 33 years | Oral immunosuppressant; Additional | Moderate |
| Female | 57 | 24 years | 18 years | Oral immunosuppressant; Steroid; Additional | Moderate |
| Female | 35 | 11 years | 3 years | Steroids (dose of 20)  Gabapentin 3000g  Tramadol 300, paracetamol; omeprazole Pain; Steroid; Additional | Moderate |
| Female | 55 | 18 years | 27 years | Oral immunosuppressant; Additional; Beta blocker | Moderate |
| Female | 35 | 3 years 7 months | 20 years | Oral immunosuppressant; Topical | Moderate |
| Female | 47 | 8 years | 20 years | Topical; Steroids; Biologic; Oral immunosuppressants; Additional; MM  Anticoagulant/thinners | Severe |
| Female | 66 | 41 years | 2 months | Additional; Steroid; Oral immunosuppressants; Pain | Moderate |
| Male | 62 | 20 years | 20 years | Biologic; Pain; Beta blocker; Oral immunosuppressant | Severe |
| Male | 36 | 18 years | 6 months | Oral immunosuppressant; Anticoagulant/blood thinner | Moderate |
| Female | 39 | 5 years 4 months | 15 years | Biologic; Steroid; Pain | Severe |

*Key:Oral immunosuppressants inc methotrexate, mycophenolate, azathioprine; Biologic inc humira, imraldi, infliximab, campath; PLEX – plasma exchange

Anticoagulant/thinners inc rivaroxaban; Topical – creams for skin lesions/ulceration; MM – magic mouthwash (triorasol – combo steroid, anti-biotic, anti-fungal)

Pain – pregabalin, oramorph, amitriptyline, codeine; Steroid – prednisolone; Additional – vitamin D supplement, omeprazole to protect stomach

Beta blockers – propranolol

** This was based on medicines taken. See the guidance from the Behçet's UK Factsheet on treatment [22], which provides guidance on treatment based on disease severity.
